# Ecological uncertainty favours the diversification of host use in avian brood parasites

**DOI:** 10.1038/s41467-020-18038-y

**Published:** 2020-08-21

**Authors:** Nicholas D. Antonson, Dustin R. Rubenstein, Mark E. Hauber, Carlos A. Botero

**Affiliations:** 1 Department of Evolution, Ecology, and Behavior, School of Integrative Biology, University of Illinois Urbana-Champaign, 505 S. Goodwin Ave., Urbana, IL 61801 USA; 2 Department of Ecology, Evolution, and Environmental Biology, Columbia University, 1200 Amsterdam Ave, New York, NY 10027 USA; 3 Department of Biology, Washington University in St. Louis, 1 Brookings Dr, St. Louis, MO 63130 USA

**Keywords:** Behavioural ecology, Evolutionary ecology, Macroecology, Coevolution

## Abstract

Adaptive responses to ecological uncertainty may affect the dynamics of interspecific interactions and shape the course of evolution within symbioses. Obligate avian brood parasites provide a particularly tractable system for understanding how uncertainty, driven by environmental variability and symbiont phenology, influences the evolution of species interactions. Here, we use phylogenetically-informed analyses and a comprehensive dataset on the behaviour and geographic distribution of obligate avian brood parasites and their hosts to demonstrate that increasing uncertainty in thermoregulation and parental investment of parasitic young are positively associated with host richness and diversity. Our findings are consistent with the theoretical expectation that ecological risks and environmental unpredictability should favour the evolution of bet-hedging. Additionally, these highly consistent patterns highlight the important role that ecological uncertainty is likely to play in shaping the evolution of specialisation and generalism in complex interspecific relationships.

## Abstract (web summary)

Nearly 17% of all bird species are hosts to obligate brood parasites like the common cuckoo. Antonson et al. show that parasite species hedge their reproductive bets by outsourcing parental care to a greater variety of host species when the rearing environment for their young is more unpredictable.

## Introduction

Species that derive fitness benefits from symbioses often vary in the number of heterospecifics with which they interact^1,2^. Although prior comparative analyses and theory suggest that predators and parasites should rely on a greater number of prey or hosts in more variable and unpredictable environments^3^, this relationship has been challenging to detect empirically because the course and pace of co-adaptation^4^ are dictated not only by environmental factors, but also by the responses of all of the species involved^5,6^. Understanding how ecological uncertainty influences the degree of specialisation in co-evolving systems is especially important in the current context of global change because climatic patterns are becoming increasingly variable and unpredictable^7–10^ and many parasites, predators, and their symbionts already face significant threats of extinction^11^.

**Fig. 1:**
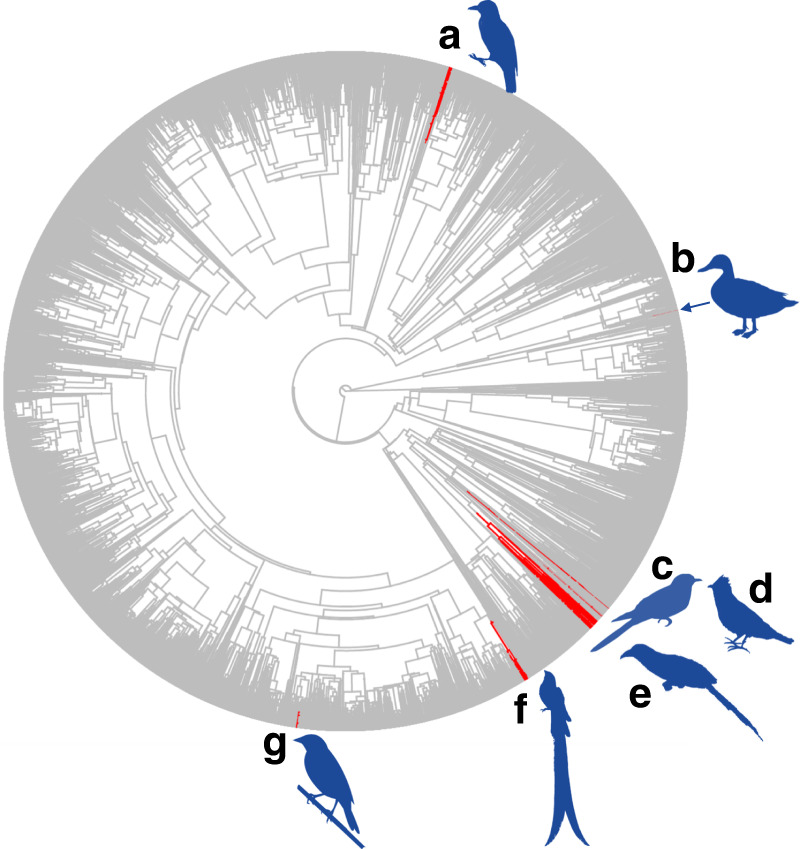
Stochastic character mapping^15^ of obligate brood parasitism (red lineages) in birds. Representative clades for each of the six known altricial and one precocial independent evolutionary origins of obligate brood parasitism in birds include **a** Indicatoridae, **b** Anatidae (arrow to character mapping), **c** Cuculinae, **d** Neomorphinae, and **e** Phaenicophaeinae, all within Cuculidae, **f** Viduidae, and **g** Icteridae. Silhouettes from N.D. Antonson (**a**, **d**) and phylopic.org with credit to Sharon Wegner-Larsen (**b**), Ferran Sayol (**c**), Lip Kee Yap (**e**), Maija Karala (**f**), and Matt Wilkins (**g**). The CC BY-SA 3.0 license can be found at https://creativecommons.org/licenses/by-sa/3.0/ and the CC BY 3.0 license at https://creativecommons.org/licenses/by/3.0/. Information on CC0 1.0 (no copyright) can be found at https://creativecommons.org/publicdomain/zero/1.0/.

Obligate avian brood parasites—species that lay their eggs in the nests of other bird species and do not raise their own young—offer a tractable and well-characterised system^12–14^ for understanding the role of ecological uncertainty in shaping species interactions. Obligate brood parasites have evolved independently multiple times across the avian phylogeny (Fig. 1)^15^ and account for roughly 1% of the more than 10,000 extant bird species. These parasites exhibit wide diversity in their degree of host specialisation^16,17^ and use nearly 17% of all bird species as hosts (See Supplementary Data 1). For example, some brood parasites, like the brown-headed cowbird (*Molothrus ater*), are generalists that can parasitise 300 different known host species, whereas others, like the yellow-throated cuckoo (*Chrysococcyx flavigularis*), parasitise only a single one. Brood parasites and their hosts also show considerable diversity in parasitic virulence, degree of co-adaptation (as measured by endogenous traits including egg mimicry), host breeding strategy, and annual reproductive output^18–22^. Evolutionarily, more recently diverged brood parasites typically specialise on fewer host species, suggesting that new parasitic lineages arise through specialised interactions with a single or a few hosts^23–25^. Thus, characterising the factors that determine host diversity in brood parasites may not only improve our understanding of resource use and host–parasite coevolution in avian systems, but also, more generally, of the evolution, maintenance, and coexistence of ecological specialisation in symbioses^26,27^.

Diversification in reproductive behaviours under environmental uncertainty has been shown across a variety of animal taxa, most notably in insects^28^ and non-parasitic (i.e., parental) birds^29^. In parental bird species, including several hosts of brood parasites, behavioural bet-hedging has been proposed as a strategy to maximise fitness in highly unpredictable environments^30^. For example, cooperatively breeding superb starlings (*Lamprotornis superbus*) that live in temporally variable savannas use bet-hedging as a strategy to spread reproductive risk over years and within seasons^31^. In brood parasites, reproductive success is dictated by their hosts’ propensity to accept parasitic eggs and by their ability to raise parasitic chicks successfully^12,32^. As such, brood parasitism is a risky endeavour, and the degree of parasite specialisation is therefore likely to be determined by factors related to the availability and quality of parenting behaviours of potential hosts^33^. Specifically, host traits such as migratory behaviour, nesting architecture, breeding phenology, chick-provisioning strategies and resilience to environmental challenges, could drive brood parasites’ host choices by dictating the success of their young. In particular, environmental uncertainty experienced through climatic oscillations could favour the spread of a brood parasite’s reproductive effort across hosts with different behavioural profiles because a wider variety of options is likely to minimise variance in parasitic offspring recruitment across years^34^. If long-term success is obtained at the expense of a lower arithmetic mean fitness, then such a brood parasitic strategy may be considered a form of diversified bet-hedging^35^.

Based on the above arguments, we predict that (1) increasing unpredictability in climatic fluctuations should lead to increased phylogenetic diversity and/or number of hosts used by brood parasites; (2) that seasonal migration by hosts and shorter breeding seasons should favour generalisation by increasing the potential for missing out on egg laying in some host species’ nests altogether; (3) that greater reliance on uniparental hosts should favour increased host generalisation by increasing uncertainty in the provisioning of brood parasitic offspring; (4) that increased reliance on cooperatively breeding hosts that exhibit alloparental care should favour increased host generalisation by increasing the difficulty of successfully parasitising host nests; (5) that parasitising hosts with more enclosed nests should increase specialisation by providing brood parasitic offspring with greater physical and antipredatory protection; and (6) that larger mean host clutch sizes should promote specialisation by making parasitic eggs less likely to stand out and be rejected (Weber’s law^36^) or by being positively correlated with hosts that are likely better able to support the demanding energetic requirements of brood parasitic chicks^22^. To control for the possibility that host diversity is partly determined by opportunity and access, we also consider the potential effects of the number of bird species co-occurring with a parasite both locally and across its range, as well as potential geographic and taxonomic biases in available natural history knowledge by including the number of studies available on each parasite (i.e., research effort) as a covariate.

Here, we use phylogenetically informed analyses to examine the extent to which symbiont characteristics and environmental conditions (as measured by the mean, variability, and predictability of precipitation and temperature cycles) influence the degree of host specialisation in avian brood parasites. Specifically, we test ecological, climatic, and behavioural predictions related to the potential sensitivity of avian brood parasites to ecological risks. Our analyses provide new insights into how brood parasites use hosts and more generally, how ecological variability, as well as its interaction with key symbiont traits, shape the evolution of behavioural specialisation in interspecific interactions.

## Results

### Multivariate correlations

As is typical in many macro-ecological analyses (e.g., ref. ^37^), we find that ecological, climatic, and behavioural variables in our dataset are strongly correlated. We therefore reduced our original set of 16 potential predictors to 8 composite variables through principal component analysis (Table 1). This conservative approach reduces the potential for multicollinearity in downstream statistical models and prevents unrealistically simplistic interpretations of observed effects. Our response variables—host species richness (number of hosts) and host phylogenetic diversity (Faith’s Phylogenetic Diversity [P.D.] values)—were also found to be positively correlated (Supplementary Fig. 1; log-log pGLS regression: Beta ± SE = 1.12 ± 0.07; *t* = 16.86; df = 81; *P* < 0.001; lambda = 0.27), suggesting that in this particular system, two potentially different types of behavioural diversification actually convey similar kinds of information.

**Table 1:** Variable loadings for a principal components (PC) analysis with varimax rotation of putative predictors of host richness and host phylogenetic diversity in altricial avian obligate brood parasites.

Variable	PC1	PC2	PC3	PC4	PC5	PC6	PC7	PC8	*h*^2^
Mean annual temperature	**0.97**	−0.05	−0.03	0.12	0.04	0.03	0.05	−0.10	0.96
Temperature predictability	**0.75**	0.46	−0.04	0.18	−0.09	0.33	−0.01	−0.17	0.95
Annual variance in temperature	**−0.66**	**−0.51**	−0.01	−0.41	0.11	−0.14	−0.01	0.16	0.92
Mean annual precipitation	**0.57**	**0.55**	−0.06	−0.10	−0.06	0.45	0.06	−0.08	0.85
CV in annual precipitation	−0.04	**−0.93**	0.04	0.20	0.09	−0.01	−0.09	0.00	0.92
Precipitation predictability	0.14	**0.86**	0.06	0.33	0.07	0.21	−0.02	−0.13	0.93
% Cooperative provisioning	0.01	−0.04	**−0.96**	−0.15	0.09	−0.03	−0.20	−0.04	0.99
% Biparental provisioning	−0.06	−0.06	**0.91**	0.03	−0.03	0.00	−0.37	0.10	0.98
% Hosts with closed nests	0.09	0.07	0.13	**0.85**	−0.16	−0.06	0.13	0.19	0.84
Local co-occurrence	0.20	0.03	0.18	**0.62**	0.36	0.49	−0.11	−0.17	0.86
% Mobile hosts	−0.45	0.13	0.01	**−0.60**	0.06	0.04	0.09	0.47	0.81
Global co-occurrence	0.06	−0.04	0.02	−0.01	**0.94**	0.12	0.01	0.02	0.91
Range size	−0.18	−0.03	−0.19	−0.10	**0.79**	−0.30	0.02	−0.18	0.82
Mean growing season	0.14	0.19	0.00	0.00	−0.09	**0.90**	0.10	−0.11	0.90
% Uniparental provisioning	0.03	0.06	−0.06	0.05	0.02	0.07	**0.98**	−0.10	0.98
Mean host clutch size	−0.25	−0.22	0.17	0.09	−0.18	−0.22	−0.21	**0.78**	0.88
Cumulative variance explained	0.17	0.33	0.45	0.57	0.68	0.78	0.86	0.93	

All variables were normalised (Box–Cox transformation) and scaled prior to this analysis and the strongest contributors to each component are highlighted in boldface type. Component labels: PC1 = Temperature harshness; PC2 = Xeric harshness; PC3 = More cooperative breeding hosts; PC4 = More protected nests, local options, and resident hosts; PC5 = More global host options; PC6 = Longer breeding seasons; PC7 = More uniparental provisioning; PC8 = Mean host clutch size.

**Fig. 2:**
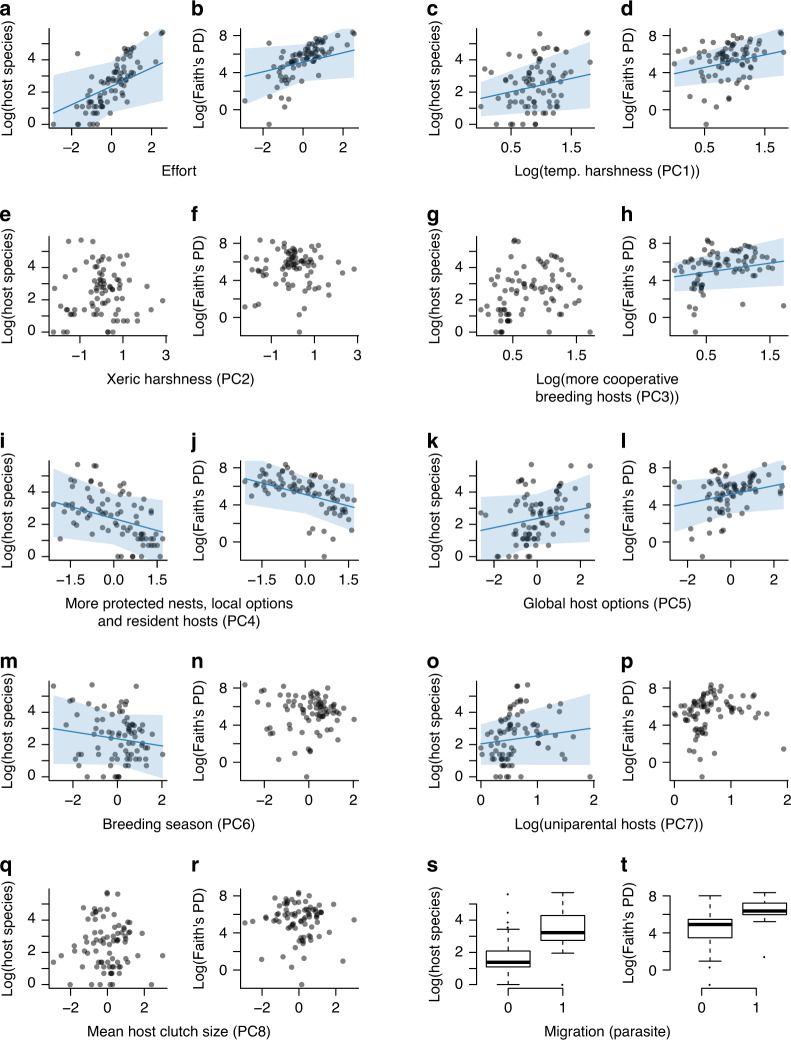
Graphical summary of the effects of ecological and climatic risk factors on avian brood parasitic host choices. Effects of research effort (**a**, **b**), temperature harshness (**c**, **d**), xeric harshness (**e**, **f**), increased reliance on cooperative breeding hosts (**g**, **h**), more protected (enclosed) nests, number of local host options, and year-round resident hosts (**i**, **j**), global number of host options (**k**, **l**), breeding season duration (**m**, **n**), increased reliance on uniparental hosts (**o**, **p**), mean host clutch size (**q**, **r**), and migratory status of the parasitic species (**s**, **t**) on the number and phylogenetic diversity of hosts of obligate avian brood parasites (*N* = 81 parasitic species). Quantitative descriptions for these correlates are presented in Table 1. Each data point represents one brood parasitic species. In the subplots of all continuous predictors for which credible intervals did not overlap zero (**a**–**d**, **h**–**m**, **o**), blue lines depict the values fitted by our Bayesian models and shaded areas depict corresponding 95% credible intervals of these relationships. In the parasite migration subplots (**s**–**t**), boxes represent the first and third quartiles, horizontal lines depict the medians, lower and upper whiskers represent 1.5 * the interquartile ranges, and dots depict outliers (differences between migration categories were not significant either for host numbers or Faith’s PD). Source data are provided as a Source Data file.

Summaries of our findings when using a minimum research effort threshold of inclusion of ten studies per parasite (see ‘Methods’) are provided in Tables 2, 3, and Supplementary Table 1, a well as depicted graphically in Fig. 2. Robustness analyses with progressively more conservative thresholds of inclusion (*K* = 10, 20, 30 or 50 studies) are presented as Supplementary Tables 2 and 3, and are largely consistent with the findings we detail below. As expected, the number and phylogenetic diversity of host species (Tables 2, 3 and Fig. 2a, b) are positively correlated with research effort.

**Table 2:** Fully simplified model of host numbers.

Parameter	Estimate	Lower 95% CI	Upper 95% CI
(Intercept)	1.294	0.508	1.991
Effort	0.570	0.280	0.864
Temperature harshness (PC1)	0.826	0.273	1.382
More protected nests, local options, resident hosts (PC4)	−0.495	−0.758	−0.222
Global options (PC5)	0.283	0.065	0.503
Breeding season (PC6)	−0.218	−0.415	−0.018
Uniparental host provisioning (PC7)	0.493	−0.008	0.983

**Table 3:** Fully simplified model of phylogenetic diversity in host use.

Parameter	Estimate	Lower 95% CI	Upper 95% CI
(Intercept)	3.163	2.202	4.016
Effort	0.514	0.157	0.867
Temperature harshness (PC1)	1.353	0.666	2.048
More multiparent provisioning (PC3)	0.980	0.368	1.582
More protected nests, local options, resident hosts (PC4)	−0.824	−1.149	−0.482
Global options (PC5)	0.476	0.192	0.764

### Ecological, climatic, and behavioural predictors of host use

After accounting for this effect, we find that some, but not all types of variability in climate are negatively associated with specialisation in obligate avian brood parasites. Specifically, brood parasites increased the number and phylogenetic diversity of their hosts (Tables 2, 3 and Fig. 1c, d) in colder environments with more variable and unpredictable temperatures. This finding is consistent with the expectation that obligate brood parasites diversify their host choices when the ideal kind of host phenotype is difficult to predict. In contrast, xeric harshness is unrelated to both the number (Supplementary Table 1 and Fig. 2e) and phylogenetic diversity of hosts (Supplementary Table 1 and Fig. 2f). We note that a similar asymmetry in the effect of different types of environmental uncertainty has been reported in studies of reproductive and life history traits, including breeding phenology and rates of infidelity and divorce^38,39^. As in those cases, the absence of a statistically detectable effect of xeric harshness suggests that the evolution of host diversification in obligate brood parasites is unlikely to be driven by temporal variation in host provisioning, which tends to be highly influenced by precipitation-related pulses in resources^40^. The lack of detectable effects of host clutch size (Supplementary Table 1 and Fig. 2q–r) favours a similar conclusion because clutch size is a well-known proxy for provisioning capability^22^.

We conclude from the above findings that the effects of environmental uncertainty on host diversification in obligate avian brood parasites are likely to be primarily related to selection for ensuring that at least some parasitic offspring are properly thermoregulated when the timing and severity of extreme temperatures are difficult to predict^41–44^. The importance of thermoregulatory stability is further supported by our finding that increased reliance on hosts with enclosed nests is associated with the use of fewer and less phylogenetically diverse hosts (Tables 2, 3 and Fig. 2i, j). Enclosed nests can provide thermoregulatory benefits by buffering harsh temperature swings during critical stages of embryo and nestling development^45,46^. However, the positive effect of increased reliance on hosts with enclosed nests could also be related to the increased frequency of enclosed nests in the tropics, where parasites have access to a larger, more diverse pool of local hosts and a greater number of year-round residents (Table 1). In other words, the effects of enclosed nests could simply be an epiphenomenon of better opportunities for finding hosts that are particularly well-suited to a parasite’s requirements^47^. Beyond these possible benefits, using more hosts with enclosed nests may also favour host specialisation due to their reduced rates of predation^46^ and their association with smaller-bodied hosts^18,25^.

Traits that influence the quality, suitability, and/or availability of hosts (e.g., migration, length of the breeding season, and host–parent provisioning strategy) are also likely to influence host use because they can affect parasitic fitness directly. Accordingly, we find that brood parasites use fewer and less diverse hosts (Tables 2, 3 and Fig. 2i, j) when a greater portion of hosts are non-migratory. Restrictions in host availability may be particularly problematic for parasites that also migrate, because when both members of a symbiotic pair leave the breeding grounds even slight differences in the timing of spring return can potentially result in highly detrimental phenological mismatches. This general interpretation is consistent with current population trends in the common cuckoo (*Cuculus canorus*)—a generalist at the species level, but a specialist at the individual level—which has experienced strong declines in recent years^48^, apparently in response to phenological mismatches driven by climate change^49,50^. As such, while individual host selection decisions may manifest in response to ecological opportunism, habitat^51^, and developmental imprinting^52^ may also constrain host use.

Along similar lines, we also find a negative correlation between breeding season duration and number of host species (Table 2 and Fig. 2m), indicating that brood parasitism can be particularly challenging when the window of opportunity for egg laying is short^32^. Specifically, stringent time constraints are likely to favour less discriminant host selection in order to facilitate the initiation of breeding activities as early as possible (see ref. ^53^). Alternatively, environments with short breeding seasons could simply facilitate host diversification by increasing host breeding synchrony^54^. Either way, the notion that opportunity helps drive the diversity of host use in obligate brood parasites is further supported by our finding that parasites with access to a greater number of potential host species also tend to parasitise a larger and more phylogenetically diverse pool of hosts (Tables 2, 3 and Fig. 2k, l).

Variation in parental provisioning strategies offers another level of risk in brood parasitic host choice. For example, multi- and alloparental provisioning could improve survival of brood parasitic young^21,55,56^ through better nestling provisioning^57,58^ and nest sanitation^59^ than uniparental care. Accordingly, we find that parasites that rely more heavily on uniparental hosts exhibit higher host richness (Table 2 and Fig. 2o). Nevertheless, it is also possible that the presence of multiple caregivers could make it harder to parasitise nests altogether^21,55,56^. Our analyses support this alternative, specifically by uncovering a positive association between increased reliance on cooperative breeding hosts and host diversity (Table 3 and Fig. 2h). A similar, albeit marginal effect is observed on host richness (Supplementary Table 1 and Fig. 2g.).

## Discussion

Our results provide clear evidence in support of theoretical predictions^3^ that obligate avian brood parasites should diversify their patterns of host use to cope with increased uncertainty in climatic conditions (in this case temperature). Obligate brood parasites do not incubate and, therefore, cannot directly manipulate the environmental conditions to which their eggs are exposed. Accordingly, we have shown that when faced with increasing thermal uncertainty, brood parasites literally ‘lay their eggs into more than one basket’, a likely case of diversification bet-hedging^28^. We have also shown that uncertainty in host availability and behaviour lead to similar outcomes. Specifically, host types are likely to differ in their ability to counter environmental threats^60^ due to variation in traits such as parental care, habitat selection, and breeding phenology^47,61,62^. Thus, a properly balanced brood parasitic portfolio of host types may ensure that a stable amount of reproductive success is achieved no matter what environmental conditions and/or individual host idiosyncrasies a parasite experiences during its lifetime^35^. Overall, our results strongly indicate that dependence on a single (or a small number of) host species may only be a viable strategy when environments are stable and predictable and host performance is more likely to be reliable. Conversely, our results also show that using a phylogenetically diverse set of hosts that exhibit a range of behavioural and life history strategies can help reduce the risk of relying on heterospecifics to raise young whenever the ideal host phenotypes are difficult to predict. Ultimately, these findings highlight the important role of ecological uncertainty in shaping the nature of symbiotic relationships and driving the evolution of specialisation and generalism.

## Methods

### Life history variables

We aggregated environmental, parasite, and host species data associated with 84 species of obligate avian brood parasites from 19 genera and five different bird families^16,17^. This species list covers ~86% of all known brood parasitic species, including members of Cuculidae, Icteridae, Indicatoridae, Viduidae, and Anatidae (Fig. 1). The list also reflects current availability of environmental and host data for brood parasites (i.e., some parasitic species have extremely poor host records and are generally data deficient).

To avoid potential sampling biases^3,25^, we followed the standard practice of including the research effort allocated to each parasite species as a predictor of the number and phylogenetic diversity of host species in our models. Research effort was measured as the number of papers published on each parasitic species according to Google Scholar (as accessed on April 27, 2020). Only species with at least ten publications and a verified list of host species (https://www.fieldmuseum.org/blog/brood-parasitism-host-lists)^63^ were included in our main analyses (*N* = 81 obligate brood parasites). An additional robustness analysis with increasingly stringent criteria for inclusion was subsequently performed to verify that our findings were qualitatively independent from this threshold (Supplementary Tables 2 and 3). A summary list of parasitic species and their total number of known and suspected hosts can be found in Supplementary Data 2.

The number and identity of the respective host species for all 84 brood parasites in our dataset were determined by aggregating data from the online repository of the Field Museum of Natural History in Chicago, IL, USA^63^ and the published records of Johnsgard^16^. Life history data for hosts included information on clutch size, migratory status, nesting architecture, and chick-provisioning strategy (Supplementary Data 1 and Supplementary Note 1). Behavioural data on brood parasites included migratory status, geographic distribution, mean number of locally co-occurring bird species, and total number of bird species within their geographic range was obtained from Birdlife International (spatial grain of these analyses was 0.5° by 0.5°)^64^. Although we acknowledge that not every co-occurring species of bird is likely to be a potentially suitable host, we chose not to limit these lists in any way (e.g., by counting only species with specific nesting architectures or habitat), because doing so would have to be based on the active behavioural choices that parasites have already made and could therefore limit our ability to see why some parasites are more behaviourally constrained than others. Migration status was coded as a binary variable where species that migrate in any portion of their range are considered migratory. As in earlier studies, the local duration of breeding seasons across the world was estimated as the average number of months in a year in which net-primary productivity (NPP) is positive (which is indicative of increased insect and plant availability^37^). For each brood parasitic species, we use the average duration of breeding season across its breeding range. NPP data ranged from 2000 to 2016 CE and was obtained from the MODIS dataset from NASA Earth Observations (provided at 0.5° resolution; http://neo.sci.gsfc.nasa.gov; accessed 18 March 2016)^65^.

### Environmental variables

We computed climate data for every locality in the world from monthly mean values of precipitation (ml/month) and temperature (°C) in a time series from 1850 to 2005 as reconstructed by Ecoclimate.org using the CCSM4 climate model^66^. Variables extracted from these time series included the mean, variability (i.e., variance for temperature and coefficient of variance for precipitation), and Colwell’s predictability index^67^.

### Statistical analyses

We used Bayesian phylogenetic mixed models (BPMM) to analyse our data as implemented in the R package *MCMCglmm*^68^. To account for uncertainty in the phylogenetic relationships between obligate brood parasitic species, we ran each model on 100 randomly selected tree topologies with Hackett’s backbone constraints^69^ downloaded from birdtree.org^70^. The resulting outputs were then combined into a single pseudo-posterior distribution and analysed accordingly. All continuous explanatory variables were Box–Cox transformed (R package: *EnvStats*)^71^, scaled, and subsequently reduced through principal component analysis (PCA) with varimax rotation (R package: *psych*^72^). Host phylogenetic diversity was estimated by averaging Faith’s P.D. values^73^ obtained from pruned host trees from the 100 randomly selected topologies using the R package *picante*^74^. Parasite migration had a high uniqueness value and was not highly loaded into any of the principal components derived from an initial PCA. To address this issue, we re-ran the PCA without it and proceeded to include it along with research effort and the resulting components of the new PCA as potential predictors in our fully parameterised model. Interactions between migration and both environmental harshness components (PC1 and PC2) were also included in the fully parameterised model to account for the possibility that migrants and residents differ in their exposure to local environmental cycles^37^. All statistical analyses were performed using the R statistical platform (R core team 2019). Sample sizes for BPMM varied depending on threshold for research effort (i.e., for *K* = 10, *N* = 81 brood parasites; for *K* = 20, *N* = 80 brood parasites; for *K* = 30, *N* = 76 brood parasites; and for *K* = 50, *N* = 73 brood parasites).

### Reporting summary

Further information on research design is available in the Nature Research Reporting Summary linked to this article.

## Supplementary information

Supplementary Information

Reporting Summary

Description of Additional Supplementary Files

Supplementary Data 1–3

## Data Availability

Brood parasite and host information can be found at https://www.fieldmuseum.org/blog/brood-parasitism-host-lists. Climate data is reconstructed from the CCSM4 climate model at www.ecoclimate.org. Net-Primary Productivity data can be found at 10.3334/ORNLDAAC/1379. Parasite trait data is publicly available from www.birdlife.org. Phylogenetic data is from www.birdtree.org. Host trait data originates from a number of publicly available datasets detailed in Supplementary Note 1. Source data are provided with this paper.

## Source data

Source Data

## Code availability

The code used to conduct these analyses and generate these figures is available as Supplementary Data 3 accompanying this paper.
