# Supplementary material for: Ecological uncertainty favours the diversification of host use in avian brood parasites
Source: Nat Commun. 2020 Aug 21;11:4185. doi: 10.1038/s41467-020-18038-y (PMC7442637; doi:10.1038/s41467-020-18038-y)
Supplement: Supplementary file 1 — Supplementary Information [file 41467_2020_18038_MOESM1_ESM.pdf]

# Supplementary Information

Ecological uncertainty favours the diversification of host use in avian brood parasites

Antonson, Rubenstein, Hauber, and Botero (2020)

**This pdf includes:**

Supplementary Figure 1
Supplementary Tables 1-3
Supplementary Note 1

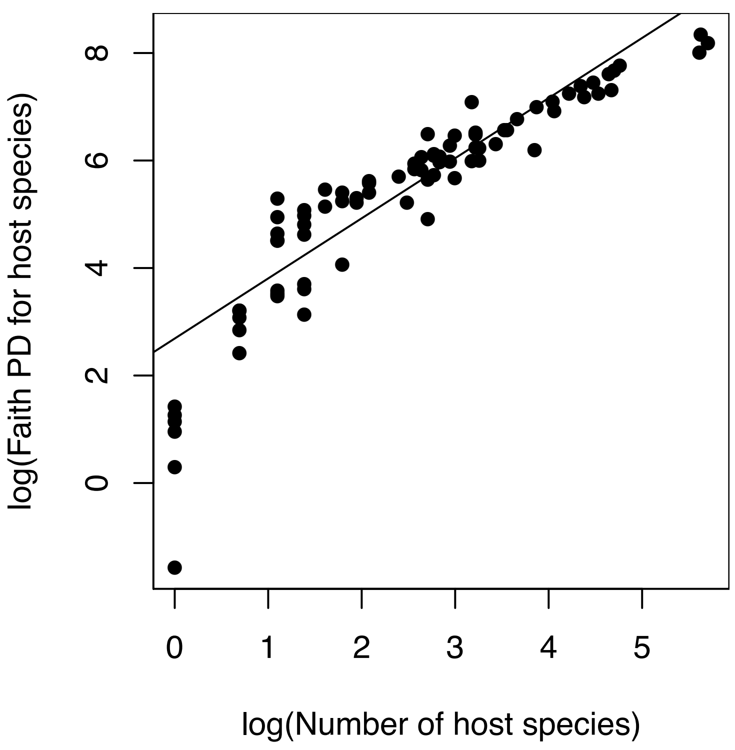


**Supplementary Figure 1.** Pattern of association between the number and phylogenetic diversity (Faith's P.D.) of host species parasitised. Source data are provided as a Source Data file

**Supplementary Table 1.** Estimates of model parameters with credible intervals that overlapped zero. Values depict the credible intervals (CI) extracted from the last model parameterization in which a given variable appeared during model reduction.

| Parameter | Estimate | Lower 95% CI | Upper 95% CI |
|---|---|---|---|
| *Models for host numbers* | | | |
| Parasite migration | 0.404 | -0.122 | 0.927 |
| Xeric harshness (PC2) | -0.090 | -0.282 | 0.103 |
| More cooperative breeding hosts (PC3) | 0.452 | -0.026 | 0.916 |
| Mean host clutch size (PC8) | -0.015 | -0.209 | 0.183 |
| Temperature harshness (PC1) * Parasite migration | 0.498 | -0.500 | 1.500 |
| Xeric harshness (PC2) * Parasite migration | 0.007 | -0.408 | 0.420 |
| *Models for phylogenetic diversity of hosts* | | | |
| Parasite migration | 0.364 | -0.357 | 1.068 |
| Xeric harshness (PC2) | -0.054 | -0.301 | 0.193 |
| Longer breeding seasons (PC6) | -0.007 | -0.268 | 0.253 |
| More uniparental provisioning (PC7) | 0.547 | -0.054 | 1.155 |
| Mean host clutch size (PC8) | -0.029 | -0.290 | 0.244 |
| Temperature harshness (PC1) * Parasite migration | 0.320 | -0.827 | 1.458 |
| Xeric harshness (PC2) * Parasite migration | 0.207 | -0.328 | 0.738 |

**Supplementary Table 2.** Robustness of the simplified model of host numbers to variation in threshold sample size for inclusion (K).

| Parameter | | Estimate | Lower 95% CI | Upper 95% CI |
|---|---|---|---|---|
| (Intercept) | | | | |
| | K = 10 studies | 1.294 | 0.508 | 1.991 |
| | K = 20 studies | 1.297 | 0.519 | 1.987 |
| | K = 30 studies | 1.579 | 0.886 | 2.210 |
| | K = 50 studies | 1.516 | 0.785 | 2.170 |
| Effort | | | | |
| | K = 10 studies | 0.570 | 0.280 | 0.864 |
| | K = 20 studies | 0.559 | 0.254 | 0.868 |
| | K = 30 studies | 0.825 | 0.494 | 1.158 |
| | K = 50 studies | 0.877 | 0.533 | 1.220 |
| Temperature harshness (PC1) | | | | |
| | K = 10 studies | 0.826 | 0.273 | 1.382 |
| | K = 20 studies | 0.830 | 0.269 | 1.395 |
| | K = 30 studies | 0.543 | 0.002 | 1.097 |
| | K = 50 studies | 0.549 | -0.001 | 1.105 |
| More protected nests, local options, resident hosts (PC4) | | | | |
| | K = 10 studies | -0.495 | -0.758 | -0.222 |
| | K = 20 studies | -0.502 | -0.770 | -0.228 |
| | K = 30 studies | -0.401 | -0.653 | -0.147 |
| | K = 50 studies | -0.388 | -0.647 | -0.121 |
| Global options (PC5) | | | | |
| | K = 10 studies | 0.283 | 0.065 | 0.503 |
| | K = 20 studies | 0.285 | 0.065 | 0.506 |
| | K = 30 studies | 0.129 | -0.089 | 0.349 |
| | K = 50 studies | 0.123 | -0.100 | 0.346 |
| Breeding season (PC6) | | | | |
| | K = 10 studies | -0.218 | -0.415 | -0.018 |
| | K = 20 studies | -0.222 | -0.421 | -0.021 |
| | K = 30 studies | -0.128 | -0.327 | 0.075 |
| | K = 50 studies | -0.107 | -0.313 | 0.100 |
| Uniparental host provisioning (PC7) | | | | |
| | K = 10 studies | 0.493 | -0.008 | 0.983 |
| | K = 20 studies | 0.493 | -0.002 | 0.986 |
| | K = 30 studies | 0.474 | -0.015 | 0.962 |
| | K = 50 studies | 0.481 | -0.008 | 0.972 |

**Supplementary Table 3.** Robustness of the simplified model of phylogenetic diversity in host use to variation in threshold sample size for inclusion (K).

| Parameter | | Estimate | Lower 95% CI | Upper 95% CI |
|---|---|---|---|---|
| (Intercept) | | | | |
| | K = 10 studies | 3.163 | 2.202 | 4.016 |
| | K = 20 studies | 3.171 | 2.220 | 4.012 |
| | K = 30 studies | 3.609 | 2.762 | 4.371 |
| | K = 50 studies | 3.512 | 2.653 | 4.292 |
| Effort | | | | |
| | K = 10 studies | 0.514 | 0.157 | 0.867 |
| | K = 20 studies | 0.441 | 0.065 | 0.818 |
| | K = 30 studies | 0.547 | 0.185 | 0.900 |
| | K = 50 studies | 0.605 | 0.240 | 0.965 |
| Temperature harshness (PC1) | | | | |
| | K = 10 studies | 1.353 | 0.666 | 2.048 |
| | K = 20 studies | 1.381 | 0.690 | 2.077 |
| | K = 30 studies | 1.063 | 0.442 | 1.707 |
| | K = 50 studies | 1.100 | 0.476 | 1.739 |
| More cooperative breeding hosts (PC3) | | | | |
| | K = 10 studies | 0.980 | 0.368 | 1.582 |
| | K = 20 studies | 0.981 | 0.376 | 1.572 |
| | K = 30 studies | 0.888 | 0.364 | 1.411 |
| | K = 50 studies | 0.900 | 0.366 | 1.425 |
| More protected nests, local options, resident hosts (PC4) | | | | |
| | K = 10 studies | -0.824 | -1.149 | -0.482 |
| | K = 20 studies | -0.866 | -1.192 | -0.524 |
| | K = 30 studies | -0.801 | -1.088 | -0.508 |
| | K = 50 studies | -0.784 | -1.074 | -0.482 |
| Global options (PC5) | | | | |
| | K = 10 studies | 0.476 | 0.192 | 0.764 |
| | K = 20 studies | 0.493 | 0.206 | 0.779 |
| | K = 30 studies | 0.318 | 0.057 | 0.582 |
| | K = 50 studies | 0.308 | 0.048 | 0.571 |

**Supplementary Note 1.** Origins of data for Antonson et al. 2020 Variables.

**Brood Parasite and Host Lists:**

1. Lowther, P.E. (2019). Brood Parasitism – Host Lists. Field Museum of Natural History, Chicago, IL. Available at https://www.fieldmuseum.org/blog/brood-parasitism-host-lists
2. Johnsgard, P. A. *The Avian Brood Parasites: Deception at the Nest*. (Oxford University Press, 1997).

**Phylogenetic Data:**

3. Jetz, W., Thomas, G. H., Joy, J. B., Hartmann, K. & Mooers, A. O. The global diversity of birds in space and time. *Nature* **491**, 444–448 (2012). https://doi.org/10.1038/nature11631
    a. www.birdtree.org

**Mean, Variability, and Colwell's Predictability for Temperature and Precipitation:**

4. Lima-Ribeiro, M. S. *et al.* EcoClimate: a database of climate data from multiple models for past, present, and future for macroecologists and biogeographers. *Biodiversity Informatics* **10**, (2015). https://doi.org/10.17161/bi.v10i0.4955

**Net Primary Productivity:**

5. ORNL DAAC 2018. MODIS and VIIRS Land Products Global Subsetting and Visualization Tool. ORNL DAAC, Oak Ridge, Tennessee, USA. Accessed Mar 18, 2016. https://doi.org/10.3334/ORNLDAAC/1379

**Brood Parasite Migratory Status, Range, and Cooccurrence:**

6. BirdLife International and Handbook of the Birds of the World. (2018). Bird species distribution maps of the world. Version 2018.1. Available at http://datazone.birdlife.org/species/requestdis.

**Mean Host Clutch Size:**

7. Del Hoyo, J., Elliott, A., Sargatal, J., Christie, D. A. & de Juana, E. Handbook of the birds of the world alive. Barcelona: Lynx Edicions (2018).
8. Myhrvold, N. P. et al. An amniote life-history database to perform comparative analyses with birds, mammals, and reptiles. Ecology 96, 3109–3109 (2015). https://dx.doi.org/10.6084/m9.figshare.c.3308127

**Host Migratory Status:**

9. Sheard, C., Neate-Clegg, M.H.C., Alioravainen, N. et al. Ecological drivers of global gradients in avian dispersal inferred from wing morphology. Nat Commun 11, 2463 (2020). https://doi.org/10.5281/zenodo.3747657
10. Del Hoyo, J., Elliott, A., Sargatal, J., Christie, D. A. & de Juana, E. Handbook of the birds of the world alive. Barcelona: Lynx Edicions (2018).

**Host Nest Architecture:**

11. Nagy, J., Hauber, M.E., Hartley, I.C. & Mainwaring, M.C. Correlated evolution of nest and egg characteristics in birds. Anim Behav 158, 211-225 (2019). https://doi.org/10.1016/j.anbehav.2019.10.015
12. Del Hoyo, J., Elliott, A., Sargatal, J., Christie, D. A. & de Juana, E. Handbook of the birds of the world alive. Barcelona: Lynx Edicions (2018).

**Host Provisioning Strategy:**

13. Cockburn, A. Prevalence of different modes of parental care in birds. Proc R Soc B: Biol Sci 273 1375-1383 (2006). http://doi.org/10.1098/rspb.2005.3458
14. Wells, M.T., Barker, F.K. Big groups attract bad eggs: brood parasitism correlates with but does not cause cooperative breeding. Anim Behav 133, 47-56 (2017). https://doi.org/10.5061/dryad.2r2j2
