# Supplementary material for: Ecological uncertainty favours the diversification of host use in avian brood parasites
Source: Nat Commun. 2020 Aug 21;11:4185. doi: 10.1038/s41467-020-18038-y (PMC7442637; doi:10.1038/s41467-020-18038-y)
Supplement: Supplementary file 2 — Reporting Summary [file 41467_2020_18038_MOESM2_ESM.pdf]

nature research

Corresponding author(s): Nicholas D. Antonson
Last updated by author(s): Jul 23, 2020

Reporting Summary

Nature Research wishes to improve the reproducibility of the work that we publish. This form provides structure for consistency and transparency in reporting. For further information on Nature Research policies, see Authors & Referees and the Editorial Policy Checklist .

Statistics

For all statistical analyses, confirm that the following items are present in the figure legend, table legend, main text, or Methods section.

n/a	Confirmed	
☐	☒	The exact sample size (*n*) for each experimental group/condition, given as a discrete number and unit of measurement
☐	☒	A statement on whether measurements were taken from distinct samples or whether the same sample was measured repeatedly
☐	☒	The statistical test(s) used AND whether they are one- or two-sided<br>*Only common tests should be described solely by name; describe more complex techniques in the Methods section.*
☐	☒	A description of all covariates tested
☐	☒	A description of any assumptions or corrections, such as tests of normality and adjustment for multiple comparisons
☐	☒	A full description of the statistical parameters including central tendency (e.g. means) or other basic estimates (e.g. regression coefficient) AND variation (e.g. standard deviation) or associated estimates of uncertainty (e.g. confidence intervals)
☐	☒	For null hypothesis testing, the test statistic (e.g. F, t, r) with confidence intervals, effect sizes, degrees of freedom and P value noted<br>*Give P values as exact values whenever suitable.*
☐	☒	For Bayesian analysis, information on the choice of priors and Markov chain Monte Carlo settings
☐	☒	For hierarchical and complex designs, identification of the appropriate level for tests and full reporting of outcomes
☐	☒	Estimates of effect sizes (e.g. Cohen's *d*, Pearson's *r*), indicating how they were calculated

Our web collection on statistics for biologists contains articles on many of the points above.

Software and code

Policy information about availability of computer code

Data collection	No code was used to collect data.
Data analysis	R version 3.6.3 with packages MCMCglmm v2.29, psych v1.8.12, picante v1.8.2, EnvStats v2.3.1, phytools v0.7-47, geiger v2.0.7, ape v5.4

For manuscripts utilizing custom algorithms or software that are central to the research but not yet described in published literature, software must be made available to editors/reviewers. We strongly encourage code deposition in a community repository (e.g. GitHub). See the Nature Research guidelines for submitting code & software for further information.

Data

Policy information about availability of data

All manuscripts must include a data availability statement. This statement should provide the following information, where applicable:

- Accession codes, unique identifiers, or web links for publicly available datasets
- A list of figures that have associated raw data
- A description of any restrictions on data availability

Brood parasite and host information can be found at https://www.fieldmuseum.org/blog/brood-parasitism-host-lists. Climate data is reconstructed from the CCSM4 climate model at www.ecoclimate.org. Net Primary Productivity data can be found at https://doi.org/10.3334/ORNLDAAC/1379. Parasite trait data is publicly available from www.birdlife.org. Phylogenetic data is from www.birdtree.org. Host trait data originates from a number of publicly available datasets detailed in Supplementary Note 1. Source data are provided with this paper.

Field-specific reporting

Please select the one below that is the best fit for your research. If you are not sure, read the appropriate sections before making your selection.

- [ ] Life sciences
- [ ] Behavioural & social sciences
- [x] Ecological, evolutionary & environmental sciences

For a reference copy of the document with all sections, see nature.com/documents/nr-reporting-summary-flat.pdf

Ecological, evolutionary & environmental sciences study design

All studies must disclose on these points even when the disclosure is negative.

Study description	Our study aimed to determine factors of ecological uncertainty that predict patterns of host use across avian brood parasites.
Research sample	We aggregated environmental and host species data from 84 species of brood parasites across 19 genera and 5 families. These data originate from publicly available sources detailed in the Data Availability statement of this paper.
Sampling strategy	We quantified research effort to ensure that it would not influence our results. Research effort was used as a predictor in our fully parameterized models. We additionally performed robustness analyses to make sure that our results were robust to increasingly more conservative research effort thresholds.
Data collection	Each author collected a different portion of the data.
Timing and spatial scale	Climate data for every locality in the world from monthly mean values of precipitation (mL/month) and temperature (oC) in a time series from 1850 to 2005 as reconstructed by Ecoclimate.org using the CCSM4 climate model (Lima-Ribiero et al. 2015). NPP data ranged from 2000-2016 CE and was obtained from the MODIS dataset from NASA Earth Observations (provided at 0.5° resolution; http://neo.sci.gsfc.nasa.gov; accessed 18 March 2016). Global co-occurrence was measured by the total number of potential host species (i.e., bird species) that a brood parasite encounters across its entire range, whereas local co-occurrence was measured as the average number of species within a parasite's range that co-occur with the parasite at a spatial grain of 0.5° by 0.5° (BirdLife International and Handbook of Birds of the World 2018). Host data was collected using publicly available host lists from the Field Museum of Natural History in Chicago, IL, USA (Lowther 2019) and the published records of Johnsgard (1997). Full potential host list is available in the supplementary information. Host behavioural and clutch size data were collected from a number of available datasets (see Supplementary Note 1 for complete details).
Data exclusions	Our sample size for the number of brood parasites analyzed was based on the current state of brood parasite research and species with fewer than 10 published records were omitted. This is intended to reduce bias that may be driven by a lower number of known hosts species for less studied brood parasites.
Reproducibility	We verify that our results are reproducible and originate from published sources that we provide in detail to facilitate replication.
Randomization	Phylogenetic analyses used randomized tree topologies.
Blinding	Authors were blind to the data collection completed by other authors.

Did the study involve field work? [ ] Yes [x] No

Reporting for specific materials, systems and methods

We require information from authors about some types of materials, experimental systems and methods used in many studies. Here, indicate whether each material, system or method listed is relevant to your study. If you are not sure if a list item applies to your research, read the appropriate section before selecting a response.

Materials & experimental systems

n/a	Involved in the study	
[x]	[ ]	Antibodies
[x]	[ ]	Eukaryotic cell lines
[x]	[ ]	Palaeontology
[x]	[ ]	Animals and other organisms
[x]	[ ]	Human research participants
[x]	[ ]	Clinical data

Methods

n/a	Involved in the study	
[x]	[ ]	ChIP-seq
[x]	[ ]	Flow cytometry
[x]	[ ]	MRI-based neuroimaging
