# Supplementary material for: Ecological uncertainty favours the diversification of host use in avian brood parasites
Source: Nat Commun. 2020 Aug 21;11:4185. doi: 10.1038/s41467-020-18038-y (PMC7442637; doi:10.1038/s41467-020-18038-y)
Supplement: Supplementary file 3 — Description of Additional Supplementary Files [file 41467_2020_18038_MOESM3_ESM.pdf]

Description of Additional Supplementary Files

File name: Supplementary Data 1
Description: Host Data

File name: Supplementary Data 2
Description: Parasite Data

File name: Supplementary Data 3
Description: R Code for Analyses
